# Review on Haploidentical Hematopoietic Cell Transplantation in Patients with Hematologic Malignancies

**DOI:** 10.1155/2016/5726132

**Published:** 2016-02-29

**Authors:** William A. Fabricius, Muthalagu Ramanathan

**Affiliations:** Division of Hematology-Oncology, Bone Marrow Transplant Service, Department of Medicine, University of Massachusetts Medical School, 55 Lake Avenue N., Worcester, MA 01655, USA

## Abstract

Allogenic hematopoietic cell transplantation (HSCT) is typically the preferred curative therapy for adult patients with acute myeloid leukemia, but its use has been reduced as a consequence of limited donor availability in the form of either matched-related donors (MRD) or matched-unrelated donors (MUD). Alternative options such as unrelated umbilical cord blood (UCB) transplantation and haploidentical HSCT have been increasingly studied in the past few decades to overcome these obstacles. A human leukocyte antigen- (HLA-) haploidentical donor is a recipient's relative who shares an exact haplotype with the recipient but is mismatched for HLA genes on the unshared haplotype. These dissimilarities pose several challenges to the outcomes of the patient receiving such a type of HSCT, including higher rates of bidirectional alloreactivity and graft failure. In the past 5 years, however, several nonrandomized studies have shown promising results in terms of graft success and decreased rates of alloreactivity, in part due to newer grafting techniques and graft-versus-host disease (GVHD) prophylaxis. We present here a summary and review of the latest results of these studies as well as a brief discussion on the advantages and challenges of haploidentical HSCT.

## 1. Introduction

A human leukocyte antigen- (HLA-) haploidentical donor is one who shares, by inheritance, precisely one HLA haplotype with the recipient and is mismatched for HLA genes on the unshared haplotype. HLA-haploidentical donors can be biological parents, biological children, full or half siblings, and collateral related donors.

Allogeneic hematopoietic cell transplantation (HSCT) is the treatment of choice with the intention of cure for some malignant and nonmalignant hematologic disorders. The hematopoietic stem cells required for this procedure are usually obtained from the bone marrow or peripheral blood of a related or unrelated donor. Historically, the best results of allogeneic HSCT have been observed when the stem cell donor is a HLA-matched sibling, but, unfortunately, an HLA-matched sibling donor (MSD) can be found in only approximately 30 percent of patients or less. For patients who lack an HLA-matched sibling, alternative sources of donor grafts can be found in suitably HLA-matched adult unrelated donors (MUD), unrelated umbilical cord blood (UCB) donors, and partially HLA-mismatched-unrelated donors (mMUD) or HLA-haploidentical related donors [1].

The major challenge of HLA-haploidentical HSCT is the intense bidirectional alloreactivity leading to high incidences of graft rejection and graft-versus-host disease (GVHD). Advances in graft techniques and in pharmacologic prophylaxis of GVHD have reduced the risks of graft failure and GVHD after HLA-haploidentical HSCT and have made this stem cell source a viable alternative for patients lacking an HLA-matched donor [2].

Historically, a MSD has been preferred over other donor sources due to improved clinical outcomes following transplant, such as improved graft failure and less GVHD, and the speed and cost-effectiveness of the search. But when a MSD is not available or suitable, the transplant center usually proceeds with an unrelated donor search and alternative donor sources (HLA-haploidentical HSCT, UCB transplant) are considered if there is an urgent need to proceed to transplantation or if a preliminary search indicates a low likelihood of finding an eight of eight allele-MUD. Unfortunately, despite an increasing number of volunteers in the unrelated donor registries, unrelated adult donor HSCT is performed in only around 35% of patients for whom an unrelated donor search has been activated [3].

## 2. Literature Review

In the past year, two large retrospective studies comparing outcomes of patients receiving haploidentical HSCT versus MSD HSCT and MUD HSCT, respectively, have been published showing promising results regarding grafting success, overall survival, and complications such as GVHD and fatal graft failure.

The first one, a large, retrospective, study published in 2015 by a Swedish group with international collaboration [9], compared data collected from 10,679 AML patients who underwent HSCT from a MSD (*n* = 9,815) and haploidentical donor (*n* = 864) between 2007 and 2012. This study showed no statistically significant difference in probability of relapse between both groups but the leukemia-free survival was superior in the MSD group when compared to haploidentical transplantation group who received either T cell-replete or T cell depleted grafts. The authors acknowledge, however, that this was a retrospective study and the different study groups were not strictly matched. Since the risk of relapse was similar in both haploidentical donor grafts and MSD grafts, we could infer a similar graft-versus-leukemia effect in both groups.

A second retrospective study that compared adults with AML who received haploidentical donor transplantation (*n* = 192), with 8/8 HLA-MUD (*n* = 1982) transplantation, showed that survival for patients with AML after haploidentical transplantation with posttransplant cyclophosphamide (PTCy) was comparable with MUD transplantation [8]. The haploidentical recipients considered in this study received calcineurin inhibitor, mycophenolate, and PTCy for graft-versus-host disease (GVHD) prophylaxis; 104 patients received myeloablative and 88 received reduced intensity conditioning (RIC) regimens. MUD transplant recipients received CNI with mycophenolate or methotrexate for GVHD prophylaxis; 1245 patients received myeloablative and 737 received RIC regimens. In the myeloablative setting, day 30 neutrophil recovery was lower after haploidentical compared to MUD transplants (90% versus 97%, *p* = 0.02). Corresponding engraftment rates after RIC transplants were however 93% and 96% (*p* = 0.25), respectively. In the myeloablative setting, 3-month acute grade 2–4 (16% versus 33%, *p* < 0.0001) and 3-year chronic GVHD (30% versus 53%, *p* < 0.0001) were lower after haploidentical, due to in vivo T cell depletion with PTCy, in comparison to MUD transplants. Similar differences were observed after RIC transplants, 19% versus 28% (*p* = 0.05) and 34% versus 52% (*p* = 0.002). Among patients receiving myeloablative and RIC regimens, there was no statistically significant difference in survival (3-year OS was 45% versus 50% (*p* = 0.38) for the myeloablative regimen group and 46% versus 44% for the RIC group (*p* = 0.71)).

In a retrospective comparative study published in 2014, Raiola et al. [6] reported data from 459 consecutive patients with hematologic malignancies, with a median age of 44 years (range of 15–71 years), who received allogeneic HSCT between January 2006 and July 2012, with grafts from MSD (*n* = 176), MUD (*n* = 43), mMUD (*n* = 43), and UCB (*n* = 105) of HLA-haploidentical family donors (*n* = 92). GVHD prophylaxis varied based on the source of donor graft: cyclosporine and methotrexate for the MSD recipients and ATG for the MUD, mMUD, and UCB recipients. PTCy, cyclosporine, and mycophenolate were used for the haploidentical transplant group.

This report showed a comparable time (16–18 days) to engraftment for all groups with the exception of UCB group that took 23 days on average to achieve absolute neutrophil count >500 (*p* = 0.001). Cumulative incidence of developing CMV antigenemia was highest in the haploidentical group: 58% in the MSD group, 60% in the MUD group, 68% in UCB group, and 74% in the haploidentical group (*p* = 0.004 for the latter). On the other hand, the cumulative incidence of transplant-related mortality (TRM) at 1000 days favored the haploidentical group: 24% for the MSD group, 33% for the MUD group, 35% for the UCB group, and 18% for the haploidentical group (*p* = 0.02). Rates of acute and chronic GVHD, relapse, and OS were comparable across all donor types.

A prospective, multicenter, nonrandomized study conducted by Wang et al. [7] between 2010 and 2013 was published in 2015 comparing results of patients with AML in complete first remission (CR1) that underwent haploidentical donor HSCT versus patients that received MSD HSCT. In this trial, 450 patients were assigned to undergo haploidentical HSCT (231 patients) or MSD HSCT (219 patients) according to donor availability. GVHD prophylaxis regimen consisted of cyclosporine A, mycophenolate mofetil, and short-term methotrexate.

The outcomes were comparable across the haploidentical and MSD HSCT groups, the 3-year disease-free survival (DFS) rate was 74% and 78% (*p* = 0.34), the overall survival (OS) rate was 79% and 82% (*p* = 0.36), cumulative incidences of relapse were 15% and 15% (*p* = 0.98), and the nonrelapse-mortality rates (NRM) were 13% and 8% (*p* = 0.13), respectively. All patients in both groups achieved donor-cell engraftment. The median time to achieve neutrophil engraftment was 2 days shorter after haploidentical HSCT (*p* = 0.004); meanwhile, platelet engraftment was achieved 3 days shorter after MSD HSCT. The cumulative incidences for grades 3 to 4 acute GVHD at 100 days were, however, 10% (95% CI, 6–14) for the haploidentical transplant group and 3% (95% CI, 1–5) for the MSD group (*p* = 0.004). The cumulative rates of severe chronic GVHD at 1 year were 12% (95% CI, 8–16) and 2% (95% CI, 0–4), respectively (*p* < 0.001), as well. In sum, the results of this study showed comparable DFS, OS, and relapse in haploidentical and MSD HSCT for AML patients in CR1. The fact that both study groups received the same GVHD regimen might explain, on the other hand, the higher incidences of acute and chronic GVHD for the haploidentical group as well as the noticeable lower incidence for the MSD group compared to prior, although retrospective, studies.

Currently, there are no published randomized studies comparing HLA-haploidentical HSCT versus UCB. However, the United States Blood and Marrow Transplant Clinical Trials Network (CTN) is conducting a phase III randomized trial of RIC and transplantation for patients with acute leukemia in complete remission or with lymphoma, comparing double UCB versus HLA-haploidentical bone marrow transplantation (BMT CTN 1101; NCT01597778). For patients who are not eligible or referred for this trial, the choice between the two graft sources remains a matter of clinician preference.

## 3. Donor Selection Criteria for Haploidentical HSCT

Most patients will have more than one HLA-haploidentical first-degree relative willing and able to donate, so the appropriate selection of the donor should follow several criteria in order to achieve the best results for a successful grafting, best graft versus leukemic effect, and to minimize graft rejection and GVHD.

A study by Kasamon et al. [10] published in 2010 showed that increasing HLA disparity between donor and recipient had no detrimental impact on the outcome of 185 hematologic malignancy patients treated with nonmyeloablative conditioning, T cell-replete bone marrow transplantation, and GVHD prophylaxis including high-dose cyclophosphamide. In this study, the presence of an HLA-DRB1 antigen mismatch in the graft-versus-host direction actually was associated with a lesser risk of relapse and increased survival.

No significant difference in overall or disease-free survival between recipients of grafts from MSD versus HLA-haploidentical donors was shown by three subsequent retrospective small studies, supporting the hypothesis that these transplantation platforms have nullified the detrimental impact of HLA mismatching on outcome. For GVHD prophylaxis, the patients received PTCy in two of these studies [6, 11] and the GIAC (granulocyte-colony stimulating factor filgrastim, intensified immunosuppression, antithymocyte globulin, and combination of peripheral blood stem cell and bone marrow allografts; see below) protocol in the other one [12].

Most selection criteria for a haploidentical stem cell donor are common to other graft types, such as ABO blood type, cytomegalovirus (CMV) serostatus of the donor and recipient, sex mismatch, and donor age and parity. There are, however, criteria that are unique to HLA-mismatched HSCT, which include donor-specific HLA antibodies, donor relationship, donor-recipient HLA mismatch, noninherited maternal antigens, and natural killer cell alloreactivity.

Absolute contraindication to the use of a specific HLA-haploidentical donor is determined by donor fitness and the presence of strong anti-donor HLA antibodies, if any, in the recipient against the donor.

## 4. Haploidentical Stem Cell Transplantation Strategies

Given the lack of large randomized comparative studies and scarcity of large prospective studies, the decision of how to plan this type of HSCT is mainly based on the expertise of practicing clinicians. Over the past few years, several strategies to HLA-haploidentical HSCT were developed. The approaches most commonly used are as follows.

### 4.1. T Cell Depletion (TCD) with “Megadose” CD34+ Cells

This modality has been associated with increased nonrelapse mortality (NRM) due to infectious complications secondary to slow immune reconstitution. It is recommended that centers, choosing this modality, have a predefined immunotherapy strategy readily available to hasten immune reconstitution and reduce the risk of infections. Initial studies of TCD required negative selection of CD3+ cells and later studies used grafts with CD34+ positive selection [13–15]. These studies using “megadose” CD34+ grafts with intensive conditioning and no additional postgrafting GVHD prophylaxis showed engraftment rates of 90 to 95% and rates of acute and chronic GVHD of <10% [16–19]. The conditioning regimen used with this approach evolved with time from TBI (8 Gy in single fraction) followed by thiotepa, cyclophosphamide, and rabbit ATG, up to newer regimens that replaced fludarabine and thymoglobulin, respectively, with cyclophosphamide and alemtuzumab [20].

Methods used to improve immune reconstitution after TCD haploidentical HSCT include CD3/CD19 negative selection [21, 22]; depletion of alpha/beta but not gamma/delta T cells [23, 24]; the infusion of cytotoxic T cell lines with viral-specificity for the prevention or treatment of viral infections [25]; and reintroduction of lower levels of both conventional and regulatory T cells [26]. Another approach infuses donor lymphocytes expressing suicide genes that could be activated in case GVHD occurs [27–29].

### 4.2. The “GIAC” Strategy

This modality is based on GCSF-stimulation of the donor with filgrastim (“G”), intensified immunosuppression posttransplantation (“I”), antithymocyte globulin (ATG—“A”) added to conditioning to help prevent GVHD and aid engraftment, and combination (“C”) of peripheral blood stem cell and bone marrow allografts. Although relatively inexpensive and not requiring significant expertise in graft manipulation, there is limited experience with this approach outside of China. When compared with high-dose, posttransplantation cyclophosphamide, GIAC appears to be associated with higher rates of acute and chronic GVHD. Conditioning is usually a modified busulfan plus cyclophosphamide regimen with antithymocyte globulin (ATG), cytarabine, and semustine (Me-CCNU).

In the original study presenting this alternative strategy [12], engraftment occurred in all 171 patients, with the cumulative incidences of acute GVHD grades 2–4 of 55% and grades 3-4 of 23%. The cumulative incidences of chronic GVHD and extensive chronic GVHD at two years were 74 and 47%, respectively. The two-year probabilities of nonrelapse mortality (NRM), relapse, and disease-free survival (DFS) were 20, 12, and 68% for standard-risk-disease patients and 31, 39, and 42% for high-risk-disease patients, respectively. Subsequent publications also indicated that this GIAC protocol could achieve complete engraftment, acceptable NRM, and favorable DFS after T cell-replete haploidentical HSCT [7, 30, 31]. Unfortunately, increased incidence of severe acute and chronic GVHD has been noted with this approach. In trying to improve these results, Italian investigators presented a report in 2013 modifying this approach through using only BM allografts and adding basiliximab [32] which allowed them to achieve a lower rate of chronic GVHD, which was 17% including both forms, limited and extensive. Furthermore, in this population, a cumulative incidence of only 5% was noted for the extensive form of GVHD. The rate of neutrophil engraftment in this approach was 93%, with only one patient having failed grafting, and the NRM was 36%.

### 4.3. High-Dose, Posttransplantation Cyclophosphamide (PTCy)

PTCy is comparatively inexpensive as it does not include graft manipulation. PTCy can also be safely used in the myeloablative conditioning setting with peripheral blood progenitor cells as a donor source. Following an initial phase I/II study and subsequent modifications to include a nonmyeloablative conditioning regimen of low-dose cyclophosphamide, and low-dose total body irradiation (TBI), a GVHD prophylaxis regimen was established consisting of posttransplant cyclophosphamide at 50 mg/kg given on each of days +3 and +4 and mycophenolate mofetil and calcineurin inhibitor tacrolimus administered for 30 and 180 days, respectively [33, 34].

According to recent publications, this strategy has shown very little negative impact of the extent of human leukocyte antigen (HLA) disparity on acute GVHD or progression-free survival (PFS) [10].

In the large retrospective study mentioned above by Ciurea et al. [8] utilizing PTCy as a GVHD prophylaxis strategy, the day 30 neutrophil recovery was slightly lower in the haploidentical compared with MUD transplants (90% versus 97%, *p* = 0.25). However, this haploidentical engraftment success rate does not vary significantly compared with the other haploidentical approaches which were about 93% in the GIAC and 90–95% in the TCD groups. The study by Ciurea et al., as well, showed no evidence of posttransplantation lymphoproliferative disease within the first posttransplant year among patients treated with PTCy [8].

Recently, a longer follow-up of a cohort of more than 370 patients showed very similar outcomes to prior studies with cumulative incidences of NRM and severe acute GVHD at six months of 8 and 4%, respectively [35]. The cumulative incidence of chronic GVHD was 13% at two years. PFS and OS rates at three years were 40 and 50%, respectively. When a disease risk index was applied to stratify across all histologies, three-year OS rates ranged from 35 to 71%. Relapse and OS estimates were comparable to those seen with HLA-matched HCST. These outcomes were also seen in two parallel studies sponsored by the Bone Marrow Transplant Clinical Trials Network (BMT CTN): a multicenter phase II trial of haploidentical HSCT (CTN 0603) and transplantation of double UCB units for high-risk hematologic malignancies after RIC.

However, higher rates of leukemia relapse have been suggested as a disadvantage in haploidentical HSCT with PTCy by data from certain studies. In the retrospective study published in 2010 by Kasamon et al. [10], higher doses of PTCy were associated with higher relapse risk, probably explained by the deleterious cytotoxic effect of cyclophosphamide on the allografts, thus impairing the antitumor effect of the latter. Five years later, in 2015, such higher risk of relapse in haploidentical PTCy regimens was also noted by Ciurea et al. [8] within the group that got RIC. Although there were no differences in survival in the RIC group, haploidentical transplantation showed a statistically significant increased risk of relapse, compared to MUD transplantation, of 58% (46–68) versus 42% (38–45), respectively. After myeloablative conditioning, a nonsignificant increase in risk of relapse was noted in the haploidentical transplantation group versus the MUD transplantation group of 44% (34–53) versus 39% (37–42), respectively.

### 4.4. Other Strategies

Other approaches are also being studied to improve the outcomes of haploidentical HSCT. One of them is the use of CD45RA depletion. This strategy has been developed over the idea that T cell depletion results in a profound and often prolonged immunocompromised state and increased risk for graft failure. Because naïve T cells are believed to be amongst the most alloreactive T cell subsets and can be identified by CD45RA expression, allogeneic HSCT using CD45RA depletion is currently being studied as an option in haploidentical donors. A recent small study [36] involved 8 children with relapsed or refractory solid tumors who were transplanted following myeloablative conditioning. Each patient received two cell products. The haploidentical donor apheresis product from the first day of collection was depleted using the CD3 Microbead reagent and from the second day was depleted after labeling with the CD45RA Microbead reagent. The products showed a median CD34 recovery of 59.2% with CD45RA depletion, compared to 82.4% using CD3 depletion. Median CD3+ T cell dose after CD45RA reduction was 99.2 × 10^6^ cells/kg, yet depletion of CD3+ CD45RA+ cells exceeded 4.5 log. CD45RA depletion also resulted in substantial depletion of B-cells (median 2.45 log). Patients received the CD3-depleted HSCT infusion on day 0 and the CD45RA-depleted infusion on day +1. All eight patients engrafted within 14 days and rapidly achieved 100% donor chimerism. No acute GVHD or secondary graft failure was observed.

Another study [37] published by the same group later in 2015 presented results from 17 patients with poor-prognosis hematologic malignancy, who received haploidentical donor transplantation with CD45RA-depleted progenitor cell grafts following a novel RIC regimen without TBI or serotherapy. The group achieved significant depletion of CD45RA+ T cells and B-cells, with preservation of abundant memory T cells, in all 17 products. Neutrophil engraftment was rapidly observed on median day +10 and full donor chimerism on median day +11 posttransplantation. There was no infection-related mortality in this heavily pretreated population, and no patient developed acute GVHD despite infusion of a median of >100 million per kilogram of haploidentical T cells.

## 5. Discussion: Advantages and Limitations of Haploidentical Donors

When compared with the other stem cell sources, the* major advantages* of the HLA-haploidentical donor option include the following:

- Increased availability of highly motivated donors: patients have an average of 2.7 potential HLA-haploidentical donors among first-degree relatives. In comparison, only approximately 30 percent of patients will have a HLA-matched sibling, and availability of an unrelated donor genotypically matched at eight of eight alleles (HLA-A, HLA-B, HLA-C, and HLA-DRB1) ranges from 16 to 75 percent depending upon the recipient's ethnic background. A recent study published in 2014 was able to determine that the likelihood of finding an available 8/8 HLA-matched donor for HSCT in the US Registry showed a wide range depending on racial and/or ethnic background. This varied from 75% for white patients of European descent versus 46% for white patients of Middle Eastern or North African descent and even lower rates were noted for black Americans of all ethnic backgrounds, whose probabilities were 16 to 19%, whereas among Hispanics, Asians, Pacific Islanders, and Native Americans, such likelihood ranged between 27% and 52% [3].
- Immediate availability: an HLA-haploidentical donor can be identified and mobilized in two weeks to one month while the time to identify and mobilize an adult unrelated donor can be longer than three months for up to 25 percent of patients.
- Adequate doses of hematopoietic stem cells (HSCs): HLA-haploidentical grafts have sufficient doses of HSCs for transplantation and of memory T cells for immune reconstitution. In contrast, the total dose of nucleated cells in a single umbilical cord blood unit may be suboptimal for engraftment in larger adults in addition to delayed immune reconstitution.
- Lower cost of graft acquisition: the costs of acquiring grafts for adult unrelated donors and umbilical cord blood are substantially higher than those of related donors.
- Immediate availability of the donor for repeated donations of HSCs or lymphocytes to treat relapse. In contrast, umbilical cord blood is a nonrecurring source of cells.
- Graft-versus-leukemia effect: for patients with high-risk acute leukemia, HLA-haploidentical HSCT may be associated with a stronger graft-versus-leukemia effect compared with HLA-matched sibling HSCT, resulting in a lower cumulative incidence of relapse [38] and an improved overall survival [39].

The* major disadvantages* of HLA-haploidentical HSCT are due to the higher frequency of host and donor T cells reactive to HLA alloantigens resulting in intense bidirectional alloreactivity [40]:

- Higher rate of fatal graft rejection.
- Severe or fatal GVHD in the absence of effective prophylactic measures.
- Attempts at T cell depletion of the donor graft and posttransplant cyclophosphamide successfully reducing the incidence of acute GVHD, but at the cost of increased incidence of graft rejection and relapse, hence without improvement of leukemia-free survival [41].
- Increased nonrelapse mortality (NRM) due to infectious complications secondary to slow immune reconstitution, mostly seen in T cell depleted strategies. Fortunately in the last decade, numerous advances in graft engineering and pharmacologic management of alloreactivity have decreased the incidences of GVHD and nonrelapse mortality and improved OS, PFS, and immune reconstitution, making this graft source an acceptable option for patients without an HLA-matched sibling or unrelated donor (Table 1) [6–8, 10, 35].

## 6. Conclusion

HLA-haploidentical hematopoietic cell transplantation is a viable treatment option for either patients who lack an HLA-identical matched sibling donor or those for whom a matched-unrelated donor cannot be found or mobilized in a timely fashion. There are no published randomized comparisons of haploidentical HSCT versus matched sibling, umbilical cord blood, or matched or mismatched-unrelated donor HSCT. Thus, the choice between these alternative graft sources depends ultimately on the urgency of the transplant and on each institutional preference.

The major advantage of haploidentical HSCT is the almost universal availability of highly motivated donors who can be mobilized in a short time at a relatively low cost. The major challenge in haploidentical transplant is from bidirectional alloreactivity that leads to graft rejection and fatal GVHD. This can be largely overcome by the use of in vivo or in vitro T cell depletion strategies, which however entails a higher risk for severe infections and relapse.

Selection of the optimal donor needs to take into consideration donor health, age, and gender, relationship to the patient, HLA mismatch in host-versus-graft and graft-versus-host directions, ABO blood type, and CMV serostatus. Other factors that can be considered include noninherited maternal antigen (NIMA) matching and natural killer cell alloreactivity as predicted by donor killer immunoglobulin receptor (KIR) haplotype matching with recipient KIR ligand.

Haploidentical HSCT regimens differ according to each center and clinician: these regimens include the use of either in vitro T cell-depleted (TCD) “megadose” stem cell graft with no pharmacologic prophylaxis of GVHD or in vivo T cell depletion using the GIAC strategy or PTCy strategy or CD45RA depletion discussed above. The excellent outcomes recently seen in haploidentical transplants have largely been made possible by the use of in vivo T cell depletion GVHD regimens such as cyclophosphamide posttransplant as well as effective immune reconstitution platforms. In summary, haploidentical stem cell transplantation, with outcomes comparable to any other graft source, is here to stay and sure to change the future landscape of transplantation. 

## Figures and Tables

**Table 1:** Comparative summary of haploidentical (HAPLO) versus matched sibling donor (MSD), matched-unrelated donor (MUD), and unrelated cord blood (UCB) hematopoietic stem cell transplantation outcomes in patients with acute myelogenous leukemia (AML).

	HAPLO	MSD	MUD	UCB
Donor availability [3]	(~50–70%)	(~15–20%)	(~20–30%)	(~50%)
Time to transplant	(~10–20 days)	(~10–20 days)	(~2–12 months)	(~2–4 weeks)
Stem cell dose (CD34+/kg) [4, 5]	~6–8 × 10^6^	~6–8 × 10^6^	~6–8 × 10^6^	~3–5 × 10^5^
Days to engraftment [6] (*p* < 0.001)	18 d	18 d	17 d	23 d
Acute GVHD, cumulative				
Grades 2–4 (*p* < 0.01) [6]	14%	31%	21%	19%
Grades 3-4 (*p* = 0.10) [6]	4%	7%	3%	1%
Grades 3-4 (*p* = 0.004) [7]	10%	3%	—	—
Grades 3-4, myeloablative conditioning (*p* = 0.02) [8]	7%	—	13%	—
Grades 3-4, reduced intensity conditioning (*p* < 0.0001) [8]	2%	—	11%	—
Chronic GVHD				
Cumulative, moderate-severe (*p* = 0.053) [6]	15%	29%	22%	23%
Cumulative, at 1 year, severe (*p* = 0.001) [7]	12%	2%	—	—
Myeloablative conditioning, at 36 months (*p* = 0.0001) [8]	30%	—	53%	—
Reduced intensity conditioning, at 36 months (*p* ≤ 0.002) [8]	34%	—	52%	—
Relapse rate				
3 y, cumulative (*p* = 0.98) [7]	15%	15%	—	—
4 y, cumulative (*p* = 0.89) [6]	35%	40%	23%	30%
Early disease (CR1, CR2) (*p* = 0.09) [6]	18%	36%	20%	24%
Advanced disease (>CR2) (*p* = 0.60) [6]	47%	47%	28%	40%
Disease-free survival				
Cumulative 3 y DFS (*p* = 0.34) [7]	74%	78%	—	—
Cumulative 4 y DFS (*p* = 0.20) [6]	43%	32%	36%	33%
Overall survival				
3 y OS (*p* = 0.36) [7]	79%	82%	—	—
4 y OS (*p* = 0.10) [6]	52%	45%	43%	34%
Relapse-related mortality [6]	26% (*n* = 24)	26% (*n* = 48)	21% (*n* = 9)	29% (*n* = 29)
Transplantation-related mortality (*p* = 0.10) [6]	18%	24%	33%	35%
Immune reconstitution: CD4+ count at posttransplant day +100 (*p* < 0.1) [6]	190/*μ*L	229/*μ*L	106/*μ*L	63/*μ*L
Cumulative incidence of CMV antigenemia (*p* = 0.004) [6]	74%	58%	60%	68%
Infection incidence at posttransplant day +100 [6]				
Bacterial	25%	23%	36%	39%
Fungal	11%	4%	14%	14%
Rate of fatal infections [6]	11% (*n* = 10)	4% (*n* = 7)	14% (*n* = 6)	17% (*n* = 18)

Data obtained from retrospective comparative studies by Raiola et al. [6], Ciurea et al. [8], and Gragert et al. [3] and a prospective study by Wang et al. [7]. The prospective study by Wang et al. [7] was the only one which used the same GVHD regimen for both the HAPLO and the MSD groups. ~: approximate; 3 y: 3 years; 4 y: 4 years; CR1: first complete remission; CR2: second complete remission; DFS: disease-free survival; OS: overall survival.
